# Urinary nerve growth factor in patients with detrusor overactivity

**DOI:** 10.1007/s11845-014-1162-8

**Published:** 2014-06-21

**Authors:** A. Korzeniecka-Kozerska, A. Wasilewska

**Affiliations:** Department of Pediatrics and Nephrology, Medical University of Bialystok, 17 Waszyngtona Street, 15-274 Bialystok, Poland

**Keywords:** Detrusor overactivity, Overactive bladder, Nerve growth factor, Urodynamics

## Abstract

**Background:**

Detrusor overactivity (DO) is one of the most frequent bladder dysfunctions in children up to the age of 18. Nowadays, the only way to confirm DO is by urodynamic investigation, which is an invasive procedure. Among the many mediators influencing bladder function, nerve growth factor (NGF) plays an important role. The present study was designed to measure urinary NGF (uNGF) levels in patients with DO diagnosed by urodynamic study in comparison with healthy controls.

**Methods:**

The investigation was conducted on 44 children, divided into two groups (24 patients with DO, 20 healthy children). Uroflowmetry was performed in all enrolled to the study and cystometry only to patients. uNGF levels were estimated in both studied groups.

**Results:**

The median uNGF level in patients with DO before treatment was higher compared with healthy controls. There were no differences between uNGF levels in patients after anticholinergic treatment and the controls. We found differences in uroflowmetry parameters between the reference group and the patients. We found correlations between uroflowmetry parameters and uNGF/cr. level.

**Conclusions:**

1. The uNGF level could be used for detecting DO in children and adolescents.
2. Measuring uNGF level is a simple, noninvasive procedure and very useful for choosing therapy in patients with DO in various clinical conditions.

## Introduction

Many factors are involved in bladder function, such as prostanoids, ATP, NO, cytokines, immunoglobulins, free oxygen radicals, nerve growth factor and physicians make several attempts to help affected patients [1]. Detrusor overactivity (DO) is the most common bladder dysfunction in children and adolescents and plays an important role in reoccurring symptoms such as: urgency, frequency with or without urinary incontinence [2]. Detrusor overactivity (DO) can complicate vesicoureteral refluxes (VURs) [3] and causes nocturnal enuresis (NE) [4]. Until now, urodynamics, an invasive procedure, was the only method for confirming DO. In recent studies, many authors focused on the role of NGF level with pathogenesis of DO [5–7].

NGF, which belongs to the neurotrophin group, is responsible for the growth and maintenance of sympathetic and sensory neurons. It plays an important role in autonomic innervations of many organs [8, 9]. Neurotransmitters, such as NGF, provide mechanisms for bidirectional communication between muscle or urothelium and nerve, leading to OAB with or without urge incontinence [9]. NGF might regulate neural function of both sensory and motor neurons [10]. Most studies concerned with NGF activity were conducted on adult populations or on animal models [5, 6, 11–13]. Changes in urine NGF levels in adult patients with DO were also observed by Kim et al. [14], Liu et al. [11, 15–17] and Kuo [18]. To the best of our knowledge, there is only one study in the pediatric population estimating NGF in children with OAB, however, in this study DO was not confirmed by urodynamic investigation in all patients [19]. Hence, we decided to assess the role of NGF in the pathogenesis of DO in children and adolescents.

The present study was designed to measure urinary NGF (uNGF) levels in patients with DO confirmed by urodynamic investigation and to compare these levels with healthy controls and before and during anticholinergic treatment. This study may answer the question whether NGF could be a biomarker of detrusor overactivity in children and adolescents.

## Methods

The investigation was conducted on 44 children, divided into two groups. The patient group included 24 children aged median 8.25 years (1.5–17). All of these children were diagnosed with DO based on urodynamic investigation. All of them were under the care of Department of Pediatrics and Nephrology and Outpatient Nephrological Clinic. The patients were examined twice: A—at the moment of DO diagnosis by urodynamic procedure (cystometry and uroflowmetry were performed and the first urine sample was obtained) and B—after 4–6 weeks of anticholinergic treatment (only uroflowmetry was performed and a second urine sample was obtained). The control group consisted of 20 healthy children aged median 11 years (3–17) with no abnormalities in urinary and nervous systems recruited to the study as children-volunteers of hospital staff.

Inclusion criteria: (1) patients: aged 1–18 years with DO in urodynamics, (2) uroflowmetry was performed in all children and cystometry was performed in patients, (3) oxybutynin treatment for 4–6 weeks after DO was diagnosed based on cystometry.

Exclusion criteria: (1) UTI in the last 2 months, (2) presence of other infections, (3) abnormalities in urinary tract or/and nervous system.

The biochemical work-up included: serum and urine creatinine (measured by Jaffe reaction); urea; and glomerular filtration rate (ml/min/1.73 m^2^) estimated by the Schwartz formula (eGFR): GFR = *k* × *H* (cm)/Lcr (mg/dl), where *k––*age-dependent coefficient (0.55 in boys under 12 years and girls at any age, 0.7 in boys over 12 years), *H—*high, Lcr—level of creatinine in serum.

The urodynamic work-up included: uroflowmetry parameters: (1) time to max flow, (2) flow and voiding time, (3) maximum and average flow rate, (4) voided volume, (5) residual urine (calculated by USG immediately after micturition); mean values of three measurements were analyzed; in cystometry: (1) detrusor pressure at urgency (Pdet urg), (2) bladder wall compliance (comp), (3) cystometric capacity (CC), 4. maximum detrusor pressure on voiding phase (max p det). Urodynamic investigations were performed after typical preparation of patients according to the ICCS rules.

In examination A the urine samples were collected during uroflowmetry before the urodynamic procedure. In examination B and reference group the samples were obtained from morning urine before control uroflowmetry. Urinary tract infections were excluded based on normal urinalysis and urine culture tests. A negative C-reactive protein (CRP) result excluded current infection.

The urine samples were taken to measure the urinary NGF (uNGF) level. Daily urine samples were frozen directly after spinning. Samples were stored in a temperature of −80 °C. Written informed consent was obtained from all enrolled subjects, subsequent to receiving full information about the study.

The uNGF levels were measured using the ELISA set (enzyme-linked immunosorbent assay). The Emax Immunoassay System (Promega, Madison, WI, USA) was used to confirm the concentration of urine NGF in both groups of children. The investigation was executed according to the manual instructions. Total uNGF levels were standardized to mg of creatinine and the results were expressed as a NGF/creatinine ratio (pg/mgcreatinine)(NGF/cr.).

Data analysis was performed using Statistica ver. 10.0 (StatSoft Inc., Tulsa, OK, USA). Normal distribution of data was tested with the Shapiro–Wilk *W* test and then statistical analysis was performed using non-parametric tests. For comparison between groups the Mann–Whitney test was used as well as the Wilcoxon and Chi square test for intra group comparisons. The Spearman test was used to assess correlations among the studied parameters. A *p* value of less than 0.05 was considered statistically significant.

The study was approved by the Ethics Committee of the Medical University of Bialystok in accordance with the Declaration of Helsinki.

## Results

The patients’ clinical characteristics are presented in Table 1. There were no differences in the age (*p* = 0.06), weight (*p* = 0.07), height (*p* = 0.1) or gender (*p* = 0.7) between the patients and the reference group.

**Table 1:** Characteristics of studied patients (with DO), healthy controls and comparison between both groups

	DO patients median (range)	controls median (range)	*p* value
Girls/boys	18/6	16/4	0.694
Age/years	8.25 (1.5–17)	11 (3–17)	0.06
Height	127.5 (77–160)	152 (100–169)	0.106
Body weight/kg	25.5 (12–64)	38.6 (12–68)	0.067
Urine osmolality	905 (752–1,058)	935 (780–1,120)	0.894
Serum creatinine (mg/dl)	0.43 (0.31–0.68)	0.52 (0.2–0.85)	0.051
GFR (ml/min/1.73 m^2^)	177.56 (129–210)	164.46 (110–330)	0.934
Urine creatinine (mg/dl)	103.51 (42.15–163.71)	106.99 (57.8–244.05)	0.122
Anticholinergic treatment (mg/kg/day)	0.2 (0.18–0.3)		
Treatment time (weeks)	5.5 (4–8)		

* *p* value <0.05

Additionally, we analyzed kidney function parameters. The results are shown in Table 1. We did not find any statistically significant difference in the serum and urine median creatinine concentrations nor in GFR. Urine osmolality was similar in the study and the reference group.

In the DO group, abnormalities in ultrasound examinations were observed in 8/24 (33 %) patients. The most frequent changes were: bladder wall thickening in 6/24 (25 %), loss of cortex/medullae differentiation in 2/24 (8.3 %). No statistically significant differences in urinary NGF/cr. level between children with and without ultrasound abnormalities were found before treatment [82.3 (3.1–223), 51.1 (3.1–239.1), respectively; *p* = 0.7] and after treatment [3.3 (0.9–6.4); 5.6 (3.8–8.5), respectively; *p* = 0.6].

LUTSs were observed in the majority of patients. The most frequent abnormality was nonmonosymptomatic enuresis. Recurrent UTIs in the DO group were found in 12/24 (50 %) patients. Detailed data are presented in Table 2. Analysis of the clinical symptoms and urodynamic findings in our patients revealed that OAB symptoms are not a reliable indicator of DO.

**Table 2:** Indications for urodynamic investigations

	Vesicoureteral reflux	Daytime incontinence	Nocturnal enuresis and daytime incontinence	Nocturnal enuresis	Recurrent urinary tract infections
Girls *n* (% of entire group)	10 (41.67)	4 (16.67)	8 (66.67)	4 (16)	10 (41.67)
Boys *n* (% of entire group)	4 (16.66)	0 (0)	4 (16.67)	2 (8)	2 (8)
Total *n* (% of entire group)	14 (58.33)	4 (16.67)	12 (50)	6 (25)	12 (50)

In further analysis, we assessed and compared uroflowmetry parameters in patients with DO (A and B) and the controls. We found statistically significant differences in most uroflowmetry parameters between the reference group and the patients, both before as well as after 4–8 weeks of treatment. However, there were no differences in maximum flow rate and average flow rate between the study and the reference group. Voided volume was larger after treatment than before but still smaller when compared with the reference group. There were no differences in the amount of residual urine. In all groups, values were in the normal range.

We evaluated Pdet urg [median 30 (7–63) cm H_2_O], CC [median 109 (50–240) ml], comp [median 11.5 (0.6–27.8) ml/cm H_2_O] and max p det [median 60 (11–149) cm H_2_O] in the cystometry test. Cystometric capacity in the study group before treatment was lower than expected for age. Similarly, bladder wall compliance was lower than normal values. All uroflowmetry and cystometry data are shown in Table 3.

**Table 3:** Uroflowmetry and cystometry parameters in studied groups of patients with detrusor overactivity (DO)

	Group A median (range)	Group B median (range)	Controls median (range)	A and B *p* value	A and C *p* value	B and C *p* value
Cystometry						
Pdet urg (cm H_2_O)	30 (7–63)					
CC (ml)	109 (50–240)					
Compliance (ml/cm H_2_O)	11.5 (0.6–27.8)					
Max p det (cm H_2_O)	60 (11–149)					
Uroflowmetry						
*T*_max_ flow (s)	4.63 (1–12)	5 (1.67–12)	7 (4–12)	0.005	<0.01	<0.01
Delay time (s)	4 (1–17.67)	3 (1–26)	2 (1–3)	0.331	<0.01	<0.01
Flow time (s)	11.5 (5–38)	12 (6.33–38)	17 (10–38)	0.004	<0.01	0.001
Voiding time (s)	12.75 (5–41)	13.5 (6.33–41)	19.5 (11–41)	0.002	<0.01	<0.01
Max flow rate (ml/s)	22.3 (8.04–46.70)	23.54 (10.73–41)	22.9 (13.3–41)	0.881	0.852	0.565
Av flow rate (ml/s)	11.9 (6.1–22.1)	12.52 (5.2–19.8)	12.8 (6.7–25.3)	0.232	0.543	0.465
Voided volume (ml)	134.34 (44.5–456)	188 (45.6–465)	219.5 (104–455)	0.008	<0.01	0.004
RU (ml)	0 (0–35)	0 (0–20)	0 (0–3)	0.173	0.215	0.289

Significant value *p* < 0.05

*A* before, *B* during anticholinergic treatment, *C* comparison among studied group and between patients and healthy controls, *Pdet urg* detrusor pressure at urgency, *Max p det* maximum detrusor pressure, *T*_max_ *flow* time to maximum flow, *Max flow rate* maximum flow rate, *Av flow rate* average flow rate, *RU* residual urine

**Fig. 1:**
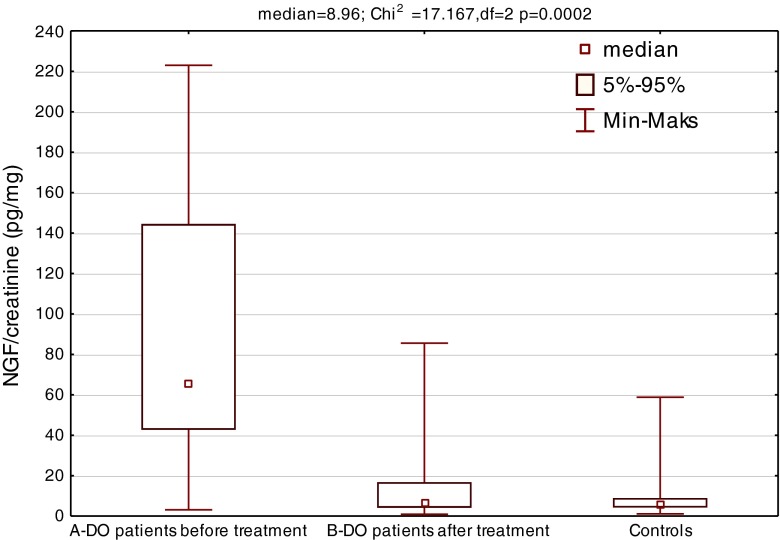
Urinary NGF/cr. median levels in patients with DO (*A* before treatment; *B* after treatment) and comparison between studied group and healthy controls. Significant value *p* < 0.05

Further statistical analysis using multiple comparisons between the groups revealed that the median value of uNGF/cr. in DO patients before treatment was significantly higher [median 65.8 (3.1–223)] when compared with the reference group [median 5.7 (1.1–58.8)] (*p* < 0.01). After a few weeks of anticholinergic treatment, uNGF/cr. decreased [median 6.1 (0.9–85.6)] and did not differ from the reference group (*p* = 0.7). The median value of uNGF level before treatment was significantly higher than after a few weeks of treatment (Wilcoxon test) (*p* < 0.01) (Fig. 1). We did not find differences in uNGF/cr. between patients with VURs and urine incontinence (*p* = 0.7).

We found correlations between uroflowmetry parameters and uNGF/cr. level. Delay time correlated positively with uNGF/cr before treatment (*R* = 0.568; *p* < 0.01). UNGF/cr. levels correlated negatively with time to max flow rate (*R* = −0.718; *p* < 0.01), flow time (*R* = −0.627; *p* < 0.01), voiding time (*R* = −0.618; *p* < 0.01), and voided volume (*R* = −0.499; *p* < 0.01). There were no correlations between uNGF/cr. levels and maximum flow rate (*R* = 0.132; *p* = 0.4) and average flow rate (*R* = 0.118; *p* = 0.6).

Additionally, we found a positive correlation between uNGF/cr. level and bladder wall compliance (*R* = 0.503; *p* < 0.01) before treatment, and a negative correlation between uNGF/cr. level and maximum detrusor pressure during voiding phase (*R* = −0.528; *p* < 0.01) before treatment.

**Fig. 2:**
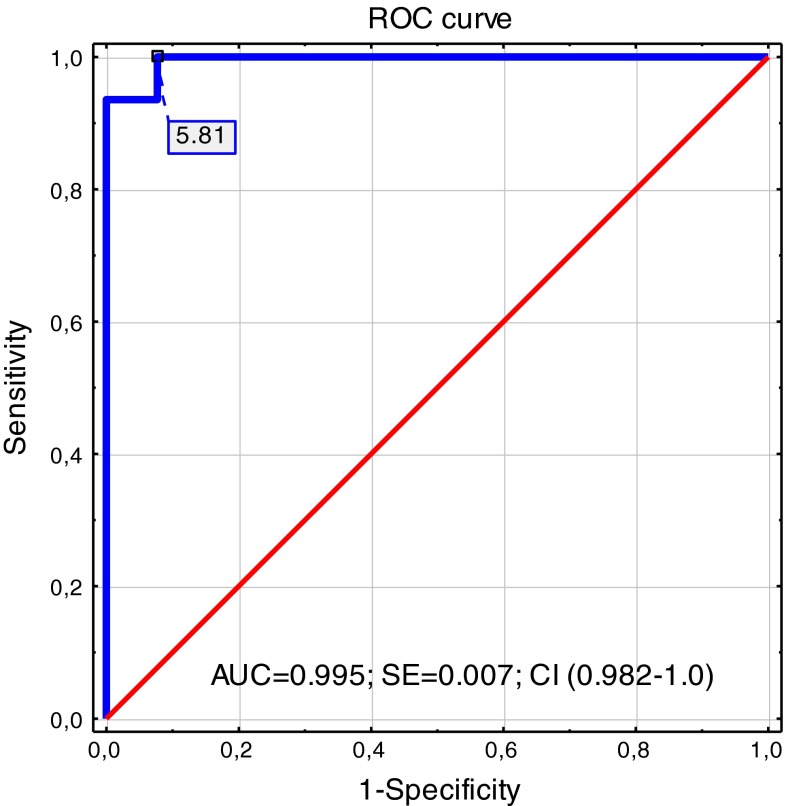
Receiver-operating characteristic curve shows sensitivity and specificity for uNGF level

We attempted to determine the cut-off value of uNGF/cr. level for the prediction of DO using a ROC curve. This analysis showed a cut-off value of >5.8 pg/mg cr. with a sensitivity, specificity, positive predictive value, and negative predictive value of 93.5, 100, 100, and 95.5 %, respectively. The area under the curve was 0.995, with a 95 % confidence interval (CI) of 0.982–1.0 (Fig. 2).

## Discussion

Detection of DO is critical in the diagnostic process of many urinary tract problems. DO can complicate vesicoureteral reflux (VUR) [3], can cause UTI [2] or nocturnal enuresis (NE) [4]. Many LUTSs appear due to DO. In part, patients with symptoms such as urgency or incontinence have normal bladder function. Diagnosis of DO is usually done using an invasive procedure, such as urodynamic investigation. However, not all patients with symptoms of OAB have urodynamically proven DO, and not all patients with DO confirmed by urodynamics have clinical OAB symptoms. Performing urodynamics is especially complicated in children up to the age of 18. Young patients can not understand why such an invasive procedure is being used. On the other hand, children cannot express their feelings exactly and the physician should confirm his suspicions through adequate tests. The situation is much more complicated when the patient has urinary incontinence and those symptoms might originate from urethral incompetence with or without detrusor contractions. The right diagnosis enables starting the appropriate treatment.

Patients with incontinence without DO had less favorable treatment results than those with DO [2].

There are a few tests, apart from urodynamic investigations, to confirm DO. Bright et al. [20] reported that bladder wall thickness could be used for detecting DO. Hubeaux et al. [21] mentioned that heart rate variability can predict DO. Last years’ study indicated that a measure of urinary NGF levels was a very simple procedure for detecting/confirming DO [5, 6, 15–17, 19]. Our results are compared with those of other authors and suggest that urinary NGF levels are associated with DO [5, 16, 19]. The cut-off value of NGF/cr. was 5.8 pg/mg and the number of false results (negative––2, positive––3) was quite low.

Assessment of uNGF levels may be valuable in such abnormalities as vesicoureteral reflux. So far, urodynamic investigation is a gold standard in detecting DO in these patients. It enables deciding on the appropriate treatment (surgical or conservative therapy). The question is whether NGF/cr. could be useful for detecting DO in patients who were treated for OAB in the past and when deterioration appeared after a temporary improvement. It might help to start the appropriate therapy immediately without urodynamic procedures.

The next very important problem is how long pharmacological therapy should last. It seems that bladder function returns to normal after a few weeks of treatment. According to Liu et al. [17] 4 weeks of therapy is an appropriate amount of time for making the decision whether therapy is adequate or not (responders or not responders).

Yokoyama [22] (based on an adult population) and Korzeniecka-Kozerska et al. [23] concluded that uNGF level was elevated in patients with neurogenic bladder independent of urodynamic findings and correlated with the final pressure in the bladder. Elevated levels of urinary NGF in DO patients may suggest that DO might be caused by neurogenic dysfunction. On the other hand Yokoyama [22] suggested that NGF levels were increased in patients who responded to resiniferatoxin (RTx) treatment. In patients with DO, it helps to change and/or choose the appropriate treatment. Our findings, like others [11, 17], show that uNGF levels decreased after anticholinergic treatment, and can suggest that DO is caused by acetylcholine release on the activation of muscarinic receptors in the bladder afferent pathways. This explains why antimuscarinic treatment is the most useful in young patients. Another study by Vijaya et al. [24] showed that urinary NGF was responsive to antibiotic therapy and women with refractory overactive bladder and elevated NGF may benefit from antibiotic treatment. We did not confirm this observation because our patients were not diagnosed with UTIs during NGF estimation, and moreover part of them received antibacterial prophylactic treatment and in spite of this we observed elevated urinary NGF level before and normalized after anticholinergic treatment.

Voiding diaries, visual analog scale and so on are good noninvasive methods for assessment of urgency, but all of them have some limitations [25]. They can not be used in infants and toddlers when the diagnosis of lower urinary tract dysfunctions is very important in many congenital abnormalities. Average daily frequency and incontinence episodes are good methods for estimating response to DO treatment but can be used in older children only. Additionally, patients with large bladder capacity, especially without incontinence, can have DO which could not be diagnosed based only on clinical symptoms. Thus, uNGF level is a good way to confirm the effectiveness of treatment in all age groups and allows for a more objective diagnosis compared with that based on subjective symptoms only. Liu et al. [17] and Finney et al. [26] claimed that antimuscarinics could be used for OB treatment during the storage phase without reducing detrusor pressure or urine flow during the voiding phase. Our current studies confirmed this effect (no differences between maximum flow rate before and after treatment) as well as correlations between uNGF level and bladder wall compliance and detrusor pressure during the voiding phase before treatment.

Our findings need to be confirmed in a further larger study to find out whether uNGF might be a DO predictor in young patients with various clinical manifestations.

Our study had several limitations. First, the study group was quite small. Second, the uNGF level was not assessed after treatment completion. We will try to continue the study, however, it is usually difficult to follow up with young patients.

In summary, the results of this current study showed that uNGF levels are higher in patients with DO when compared with the reference group, and decreased after anticholinergic treatment and correlated with urodynamic findings.

Further studies are necessary to answer the question whether uNGF could be used for DO detection in children and adolescents. Measurement of uNGF level is a simple, noninvasive procedure for choosing therapy in patients with DO in various clinical conditions.

## Abbreviations

CC: Cystometric capacity
CRP: C-reactive protein
Comp: Compliance of the bladder wall
DO: Detrusor overactivity
EMG: Electromyography
GFR: Glomerular filtration rate
MMC: Myelomeningocele
NB: Neurogenic bladder
OAB: Overactive bladder
UTI: Urinary tract infection
VUR: Vesicoureteral reflux
